# Quality Control and Safety Assessment of Online-Purchased Food Supplements Containing Red Yeast Rice (RYR)

**DOI:** 10.3390/foods13121919

**Published:** 2024-06-18

**Authors:** Celine Vanhee, Bram Jacobs, Michael Canfyn, Svetlana V. Malysheva, Marie Willocx, Julien Masquelier, Koenraad Van Hoorde

**Affiliations:** 1 Service Medicines and Health Products, Scientific Directorate of Chemical and Physical Health Risks, Sciensano, J. Wytsmanstraat 14, B-1050 Brussels, Belgium; 2 Service of Foodborne Pathogen, Scientific Directorate of Infectious Diseases in Humans, Sciensano, J. Wytsmanstraat 14, B-1050 Brussels, Belgium; 3 Toxins Unit, Service of Organic Contaminants and Additives, Scientific Directorate of Chemical and Physical Health Risks, Sciensano, Leuvensesteenweg 17, B-3080 Tervuren, Belgium

**Keywords:** red yeast rice, targeted LC-HRAM MS/MS, citrinin, mycotoxins, bacterial contamination, adulteration

## Abstract

Dietary supplements containing red yeast rice (RYR), a fermentation product of the fungus *Monascus purpureus* grown on white rice, remain popular in Europe as proclaimed cholesterol-lowering aids. The cholesterol-lowering effects are due to the occurrence of monacolin K, which is often present as a mixture of monacolin K lactone (MK) and as monacolin K hydroxy acid (MKA). MK is structurally similar to the cholesterol-lowering medicine lovastatin. Recently, due to safety concerns linked to the use of statins, the European Commission prohibited RYR supplements with a maximum serving exceeding 3 mg of total monacolins per day. Moreover, the amount of the mycotoxin citrinin, potentially produced by *M. purpureus*, was also reduced to 100 µg/kg. Evidently, manufacturers that offer their products on the European market, including the online market, must also be compliant with these limits in order to guarantee the safety of their products. Therefore, thirty-five different RYR supplements, purchased from an EU-bound e-commerce platform or from registered online pharmacies, were screened for their compliance to the European legislation for citrinin content and the amount of total monacolin K. This was conducted by means of a newly developed LC-MS/MS methodology that was validated according to ISO 17025. Moreover, these supplements were also screened for possible adulteration and any contamination by micro-organisms and/or mycotoxins. It was found that at least four of the thirty-five RYR supplements (≈11%) might have reason for concern for the safety of the consumer either due to high total monacolin K concentrations exceeding the European predefined limits for total monacolins or severe bacterial contamination. Moreover, three samples (≈9%) were likely adulterated, and the labeling of six of the seventeen samples (≈35%) originating from an EU-based e-commerce platform was not compliant, as either the mandatory warning was missing or incomplete or the total amount of monacolins was not mentioned.

## 1. Introduction

According to the World Heart Association, high levels of low-density lipoprotein (LDL) cholesterol are responsible for 4.4 million deaths per year, which represents 7.8% of all deaths worldwide [1]. High levels of LDL cholesterol can lead to plaque buildup in arteries which could result in heart diseases, including myocardial infarction and atherosclerotic cardiovascular disease, or stroke [2,3,4]. Often, high levels of LDL cholesterol are treated with lifestyle changes and medicines, including the popular drug class “statins” [3,5]. Alternatively, in recent decades, dietary supplements containing red yeast rice (RYR) have emerged in the USA and Europe and remain very popular nowadays as proclaimed cholesterol-lowering aids [3,6,7]. RYR, a fermentation product of the fungus *Monascus purpureus* grown on white rice, has been used in Eastern Asia as a traditional medicinal product to treat, among other things, lipid metabolism dysfunction and to improve blood circulation [8,9,10]. The cholesterol-lowering effects are due to the presence of monacolin K, the most abundant monacolin in RYR [11]. Monacolin K (MK_total_) exists in two forms, the monacolin K lactone form (MK), structurally identical to a statin drug called lovastatin, and hydroxyacid monacolin K (MKA) [12]. Further study has found that both lovastatin and MK act as a prodrug, and MK’s lipid-lowering effects depend on its conversion into active MKA. This process, similarly to that of lovastatin, requires the consumption of carboxylases, which may cause liver and kidney damage [13]. Although lovastatin and statins, in general, are highly effective and considered generally safe for most people, their use has also been linked to headaches, nausea, rash, decreased libido, muscle pain, new onset of diabetes, and psychiatric adverse effects in some people [12,13,14,15,16,17,18]. Consequently, dietary supplements containing lovastatin or MK are considered “an unapproved drug” in the USA and are therefore illegal [19]. However, RYR, when devoid of MK, remains commonly available as an over-the-counter product [20]. Europe, on the other hand, did not ban MK-containing RYR products, but in 2022 limited the total amount of monacolins that could be present in RYR-containing dietary supplements to a maximum of 3 mg per day [21]. As a result, the dietary supplement market shifted from pure RYR powder to products containing a mixture of RYR and plant extracts and/or coenzyme Q10 so that they might continue to use the cholesterol-lowering health claim. In addition to the potential presence of monacolins, RYR is known as the food product with the highest incidence of severe contamination with the nephrotoxic mycotoxin citrinin, potentially also produced by *M. purpureus* [7,22,23,24]. Initially, the limits for citrinin present in RYR supplements were set at 2000 µg/kg [25]. However, in 2019, the European Commission lowered the maximum tolerated levels of citrinin in RYR-containing dietary supplements to 100 µg/kg, due to safety concerns [26]. It stands to reason that the total amount of monacolins and specifically the amount of MK_total_ and citrinin are pivotal quality control parameters which should be assessed at the end of the production chain and in the finished products to ensure the safety of the consumer. Additionally, the occurrence of other mycotoxins (e.g., ochratoxin A, deoxynivalenol, or aflatoxins) and/or mycotoxin-producing fungi, including *Aspergillus* spp. and *Penicillium* spp., are important to evaluate, as severe contaminations have been described in the past [27]. These contaminations were either a result of improper storage or adulteration by “lovastatin” produced by *Aspergillus* spp., as this production is generally considered a cheaper option. Moreover, a recent paper described the occurrence of simvastatin-containing RYR supplements [22]. These findings are quite surprising and might also be the result of some form of adulteration. The latter is not so surprising as the European Know-X database on falsified medicinal products, including medicines in disguise, reported the occurrence of synthetic statins in dietary supplements. 

This study assessed different quality parameters that could play an important role in guaranteeing the safety of RYR dietary supplements. A total of thirty-five RYR supplements, either purchased from an EU-bound e-commerce platform or from registered online pharmacies, were screened for the presence of different monacolins, citrinin, and non-RYR-related statins. Next, the amount of MK_total_ and citrinin was simultaneously determined by a novel methodology employing liquid chromatography coupled to high-resolution accurate mass (LC-HRAM) mass spectrometry (MS). The methodology was subsequently also validated according to ISO 17025 [28], by employing accuracy profiles [29]. Our results demonstrated that three supplements exceeded the limits set by the European Commission on monacolin content. Next, the supplements were also analyzed for the possible occurrence of adulteration with lovastatin by analyzing the ratio of MK to MK_total_ and by verifying the stable carbon isotope ratios (δ^13^C), as conducted previously [30,31]. Finally, the amount of other mycotoxins and the bacterial load were assessed. None of the samples contained quantifiable amounts of one of the four main aflatoxins (B1, B2, G1, G2), and only one sample contained 5 ng/g of ochratoxin A, which does not represent a reason for concern as this concentration is below the limit set for similar food materials, such as spices, which are also taken in similar daily mass intake [32]. However, bioburden analysis showed that one sample was severely contaminated with an *Acinetobacter* species that was able to grow on bile acid medium, indicating that these bacteria would end up in the intestinal system. For this particular species, limited data are available on the effects these bacteria might have in the gut. The presence of more than 2.5 × 10^3^ colony forming units (CFU) makes this sample non-compliant with the quality requirements set out by the United States Pharmacopoeia (USP) for dietary supplements and those by the European Pharmacopoeia for herbal medicinal products [33,34]. Although the latter is specifically dedicated to medicinal products, very often the described limits are used as quality control limits for dietary supplements.

## 2. Materials and Methods

### 2.1. Sample Set

A total of 35 dietary supplements branded to lower cholesterol and listed as containing RYR were purchased online from either online pharmacies that are licensed by Belgian health authorities or from an EU-based e-commerce platform. Their country of origin and the ingredients listed on the label are mentioned in Supplemental Table S1. All samples were stored at room temperature (15 to 25 °C), protected from light, and analyzed before their expiration date.

### 2.2. Solvents, Reagents, and Standard Solutions

**Figure 1:**
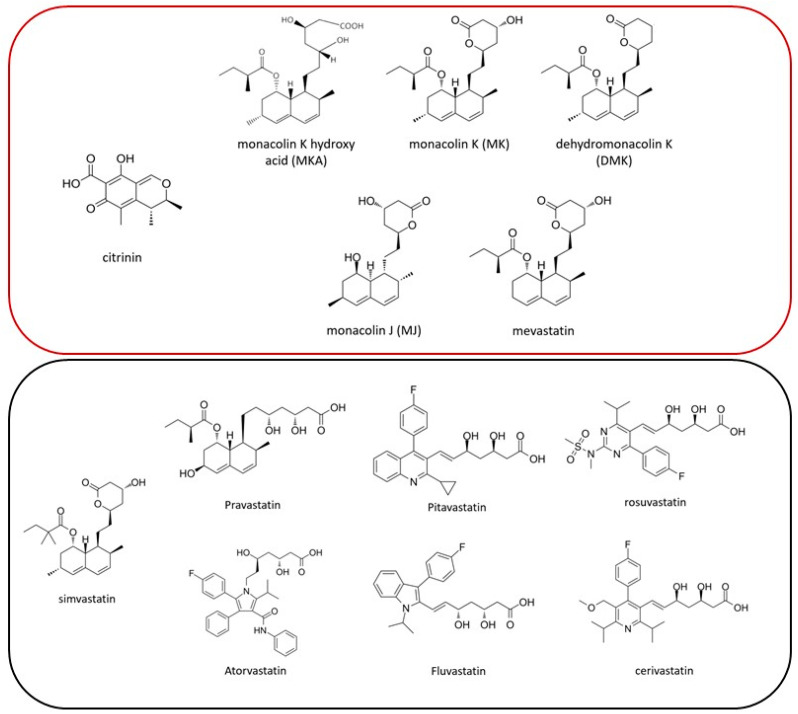
Chemical structures of the different molecules incorporated into the screening methodology. The molecules present in the upper rectangle (red) are known to potentially be present in RYR supplements, while the ones present in the lower rectangle (black) are not, as they are either generated synthetically or produced by organisms other than *M. purpureus*.

Mass spectrometry (MS)-grade acetonitrile (purity 99.8%) and formic acid (purity > 99%) were purchased from Thermo Fisher scientific (Waltham, MA, USA). Water was generated using a Milli-Q Gradient A10 system (Millipore, Billerica, MA, USA). Reference standards of the different monacolins present in RYR (monacolin K, hydroxyacid monacolin K, mevastatin, dehydrolovastatin, lovastatin diol lactone, or mevastatin J), and non-RYR statins, for simplicity termed “synthetic statins” hereafter (e.g., simvastatin, pravastatin, and the different synthetic statins, including atorvastatin, cerivastatin, fluvastatin, pitavastatin, and rosuvastatin), were acquired from Merck (St. Louis, MO, USA) (see Figure 1). The stable labeled internal reference standard (SIL) of U-[^13^C13]-Citrinin (10.14 ± 0.12 µg/mL) was bought from Romer labs (Getzersdorf, Austria), while the SILs for MK and lovastatin-d3 (purity = 90.5%) and the SILs for MKA and lovastatin-d3 hydroxy acid sodium (purity = 97%) were purchased from Toronto Research Chemicals (North York, Ontario, Canada). The initial standard stock solutions of 2 mg/mL of either reference standard and SIL MK or MKA were prepared in methanol prior to dilution to a working stock solution of 100 μg/mL in methanol. Both stock solutions were stored at −20 °C and kept for 6 months. 

In order to determine the limit of detection of the different molecules, serial dilutions were made from the standard stock solutions in methanol. For the generation of the calibration curves, standard stock solutions were diluted into 6 different concentrations in methanol. However, for validation of the screening and quantification method, the reference standards were diluted in the chosen matrix and extracted with methanol (see further). 

Reference standards of the different mycotoxins (Aflatoxin B1, purity = 99.5%; Aflatoxin B2, purity = 99.7%; Aflatoxin G1, purity = 98%; Aflatoxin G2, purity = 97.1%; Ochratoxin A, purity = 99%; T2, purity = 98.8%; Deoxynivalenol, purity = 98.3%; Fumonisin B1, purity = 97.6%; Fumonisin B2, purity = 99%; Fumonisin B3, purity = 98.5%; Zearalenone, purity = 99.7%) and related internal standards (^13^C-Aflatoxin B1, purity = 99%; ^13^C-Aflatoxin B2, purity = 99%; ^13^C-Aflatoxin G1, purity = 99%; ^13^C-Aflatoxin G2, purity = 99%; ^13^C-Ochratoxin A, purity = 98.7%; ^13^C-T2, purity = 98.8%; ^13^C-HT2, purity = 98.9%; ^13^C-Deoxynivalenol, purity = 99%; ^13^C-Fumonisin B1, purity = 96.3%; ^13^C-Fumonisin B2, purity = 98.7%; ^13^C-Fumonisin B3, purity = 98.7%; ^13^C-Zearalenone, purity = 98.8%) were acquired from Romer labs (Getzersdorf, Austria) and a reference standard for HT-2 (purity = 99%) was obtained from Cfm Oskar Tropitzsch GmbH (Marktredwitz, Germany).

### 2.3. Targeted Screening Methodology for Natural Statins (Monacolins) and Synthetic Statins

Sample preparation: The majority of the dietary supplements were present in the form of capsules or tablets. In the case of capsules, at least 10 items of each sample were weighed and then opened to mix and homogenize their content prior to analysis. The samples consisting of tablets were also weighed and 10 units were ground by mortar and pestle. About 250 mg of dry finely ground and powdered sample was weighed into a cylinder, resuspended in 10 mL of methanol, sonicated for 15 min, and passed through a 0.2 μm polytetrafluoroethylene (PTFE) filter before injection into the LC-HRAM-MS systems. For the two samples consisting of soft gel capsules, the interior of at least 5 capsules was mixed by vortexing and 250 mg of jelly was weighed into a cylinder and resuspended in 10 mL of methanol, followed by sonication and filtration prior to injection into the LC-HRAM-MS systems.

Instrumental settings: High-resolution accurate mass (HRAM) tandem MS (MS/MS) analyses were carried out on a Thermo Scientific™ Vanquish™ ultra-high-performance liquid chromatography (UHPLC) system coupled to a Q Exactive™ focus orbitrap mass spectrometer (Thermo Fisher Scientific, Bremen, Germany). 

The optimized LC methodology, employing an Acquity UPLC BEH C18 column (100 mm × 2.1 mm, 1.7 μm particle size) (Waters, Milford, MA, USA), was as follows: isocratic elution for 1 min at 5% mobile phase B (0.1% formic acid in acetonitrile) at a constant flow rate of 0.4 mL/min and a column temperature of 45 °C, followed by a steep increase to 65% B and gradient to 95% B in 5.9 min, which was kept for 1.5 min prior to a final re-equilibration step of 1.5 min with 95% mobile phase A (0.1% formic acid in water), resulting in a total run time of 10 min. 

The high-resolution accurate mass analysis was performed on a Q-Exactive Focus Orbitrap MS (Thermo Fisher scientific, Bremen, Germany), equipped with a heated electrospray ionization (ESI) source operating in positive ion mode. The optimized tune method was set as follows: the flow rate of sheath gas (nitrogen, purity ≥ 99.99%) and auxiliary gas (nitrogen, purity ≥ 99.99%) was set at 30 and 10 arbitrary units, respectively; the temperature of the capillary was set at 320 °C; the voltage of the spray was 3.2 kV; and the S-lens RF level was set at 60 V. The MS scan data were acquired at a resolving power of 35,000 (at *m*/*z* 200). The MS^2^ data, with a resolving power of 17,500, were obtained with the use of parallel reaction monitoring (PRM) scanning mode for the identification of metabolites. The collision energy for the targeted precursor ion-triggered collision-induced dissociation (CID) was adjusted to 40%. The monitored precursor *m*/*z* values (inclusion list) are listed in Supplemental Table S2. All data were collected, acquired by using the Thermo Xcalibur 4.0 software, and processed by TraceFinder 5.1 software (Thermo Fisher Scientific, Bremen, Germany). A compound was considered present provided that the difference in retention was less than or equal to 0.5 min (compared with the retention time of the reference standard of this compound), the *m*/*z* of the precursor ion was equal to the one obtained with the reference standard (error tolerance: 5 ppm), and the MS/MS spectrum corresponded to the fragment ions observed for the reference standard (fragment ions and their relative intensities are given in Supplemental Table S2). The acceptable relative errors on the relative intensities of the fragment ions were the following: for relative intensities between 10 and 40:30%; between 40 and 60:25%; and above 60 a relative error of 10% was deemed acceptable. 

Validation of the screening methodology: According to the International Council for Harmonization (ICH) guidelines, the developed methodology should distinguish citrinin, the different monacolins, and statins from each other and from the matrix ingredients [35]. This can be achieved by combining the two separation mechanisms of LC and MS^n^. The specificity and the selectivity of the utilized LC-MS^n^ method must be demonstrated for a certain concentration level, and the screening detection limit (SDL) for which the different compounds (citrinin, the different monacolins, and synthetic statins) must be correctly identified in all of the samples. The screening detection limit (SDL) of the components was experimentally determined by serial dilutions in 3 different matrices and corresponded to the lowest concentration for which a signal-to-noise (S/N) ratio (peak to peak) reached a value equal to or exceeding 3.3 and where the fragment ions were still present in the MS^n^ spectrum (see Supplemental Table S2). Matrix 1 contained a mixture of different herbal extracts (*Olea europaea*, *Allium sativum*, and *Allium ursinum* extract). Matrix 2 contained a mixture of vitamin C and different herbal extracts (*Citrus bergamia* and *Cynara scolymus* extract). Matrix 3 consisted of different vitamins (vitamins B1, C, E, and K), coenzyme Q10, and different herbal extracts (*Pterocarpus*, *Olea europaea*, and *Vitis* extract).

### 2.4. Quantification of Citrinin and Monacolin K

Sample preparation: The samples were prepared as mentioned for the screening methodology but were spiked with SIL internal standards prior to injection into the LC-HRAM-MS system. For quantification purposes, serial dilutions were made until a concentration within the interval of the calibration line was obtained. All quantifications were performed in triplicate.

Instrumental settings: The same instrumental conditions as those employed for the screening methodology were utilized except for a difference in the inclusion list, as the SIL internal standards need to be monitored, while this is not the case for the synthetic statins (see Table 1). 

Validation of the quantification methodology: Validation of the monacolin quantitation method was performed based on ICH guidelines [35] and the total error approach [36,37], as was performed previously by our research group when analyzing food supplements [38,39]. Briefly, the selectivity and specificity of the method were assessed by comparing the 3 blank matrices and those same matrices spiked with the different compounds. In the case of MK and MKA, a red yeast rice sample devoid of these two substances was also tested. Next, the limit of detection (LOD), the lower limit of quantification (LLOQ), and the upper limit of quantification (ULOQ) were determined and are summarized in Table 1. The linearity was assessed for the compounds of interest and adequate linearity was achieved as the regression coefficient, (R^2^) ≥ 0.99, and Mandel’s fitting deemed a linear relationship more suitable than a quadratic one [40]. 

Next, potential matrix effects were evaluated by comparing the obtained amounts of the different compounds at their LLOQ and ULOQ. All injections were performed in quadruplicate and did not exceed acceptance limits of 100% ± 10%, indicating no significant matrix effect (see Table 2). 

Trueness, precision, and accuracy were determined by means of the total error approach. This approach estimates the total error (TE) by combining the systemic error (bias) and the random error (intermediate precision) to determine the difference between the observed result and the true value. In other words, the highest error of an analytical method can be estimated. The TE (%) is calculated as bias (%) + 1.65 × intermediate precision (%). The factor 1.65 implies that 95% of the results will fall within the TE limit, given a Gaussian distribution. 

For this experiment, blank samples, consisting of the most complex matrix, Matrix 3, were spiked with citrinin, MK, MKA, and the different SILs at 5 different concentration levels within the linear range, and were analyzed for at least three consecutive days. Each day, a calibration curve was generated for each component and for six concentration levels. The corresponding concentrations were calculated from the measured peak areas using the calibration curve. These calculated concentrations (i.e., the 5 concentration levels) were used to determine, for this concentration range and this compound, the applicability of a linear relationship between the measured and theoretical concentrations, the trueness, precision (repeatability and intermediated precision), and accuracy, by means of a validated Excel spreadsheet that was used previously [38,39]. The linearity of the generated method was deemed acceptable as the obtained R^2^ values were above 0.999 and the *p*-values of the lack of fit (LOF) test were above 0.05. Moreover, the trueness, a measure for the systematic error of the method, and the precision were calculated for each component, as can be seen in Supplemental Table S3. Finally, the accuracy, which takes into account the total error of the test results, is represented by the β-expectation tolerance limits. Taking into account that dietary supplements have a more complex matrix than pure active pharmaceutical ingredients (APIs), the acceptance limits were set at [−20%; 20%]. The β-expectation tolerance limits did not exceed the acceptance limits as the lowest and highest obtained values did not surpass these ±20% (see Supplemental Table S3 and Supplemental Figure S1). 

### 2.5. Elemental Analyzer–Isotope Ratio Mass Spectrometry (EA-IRMS) 

Approximately 10 g of homogenized powdered sample was sent to the Fondazione Edmund Mach Italy, where it was subjected to preparative HPLC, lyophilized, and analyzed in duplicate via EA-IRMS in accordance with Perini et al. [31].

### 2.6. Bioburden Determination and Identification of the Encountered Micro-Organisms

Bioburden testing was performed as mentioned in [41,42]. The encountered bacteria were isolated on trypto-casein-soy agar for further analysis, while the encountered fungi were isolated on Sabouraud dextrose agar prior to downstream identification. Both bacterial and fungal isolates were analyzed by means of a Matrix-Assisted Laser Desorption Ionization–Time of Flight mass spectrometry (MALDI-TOF MS) Biotyper^®^ (Bruker Daltonics, Bremen, Germany) as performed previously [41]. The generated data were processed using the MALDI Biotyper 3.0 software (Bruker Daltonics, Bremen, Germany). Only hits with log(score) values equal to or higher than 2.00 were considered as high-confidence identification at the species level. In case of no hits with a sufficient log(score), hits with log(score) values between 1.70 and 2.00 were reported at the genus level. Hits with log(scores) below 1.7 were considered not identified. 

### 2.7. Mycotoxin Determination

The different mycotoxins present in the sample were extracted with a modified QuECHERS method [43]: a solid–liquid extraction followed by a MgSO_4_ (purity 98.6%)/NaCl (purity 99.9%)-induced phase separation in which the mycotoxins are concentrated. Next, the samples were submitted to targeted LC-MS/MS analysis. This methodology, validated according to ISO 17025, has been accredited by the national accreditation body and was utilized successfully upon participation in proficiency tests [44]. Briefly, 17.5 mL of an extraction solution (acetone/water/isopropyl alcohol/acetic acid (43:42:14:1, *v*/*v*/*v*/*v*)) was added to 4 g of the homogenized sample and mixed for 1 h on a rotating wheel before the addition of the salts. Next, the sample was centrifuged, the supernatant was taken, and a certain amount of SIL mycotoxin (for each analyzed mycotoxin) was added prior to injection and targeted analysis. This final extract was analyzed by HPLC-MS/MS using a Xevo TQ-S triple quadrupole MS coupled to an Acquity UPLC H Class (Waters, Milford, MA, USA). Analyte separation was performed using a Kinetex XB-C18 HPLC column (2.6 µm, 100 × 4.6 mm, Phenomenex) at 40 °C. The mobile phase comprised phase A (Milli-Q water) and phase B (MeOH). In both phases, 10 mM ammonium formate and 0.1% formic acid were added. The flow rate used was fixed at 1 mL/min, and the applied gradient elution program was as follows for mobile phase B: 0 min, 2.5%; 2 min, 2.5%; 12.5 min, 100%; 14 min, 100%; 15 min, 2.5%; 18 min, 2.5%. The sample manager temperature was fixed at 10 °C and the sample injection volume was 1 µL. MS analysis was performed using an electrospray ionization source operating in positive mode. The optimized configurations for the MS instrument were as follows: source temperature 150 °C, desolvation temperature 300 °C. Cone and desolvation gas flows were set at 150 and 800 L/h, respectively. The capillary voltage was set at 3.0 kV. The collision gas flow corresponded to 0.15 mL/min with argon as collision gas, and the source offset was 50 V. The different transitions for the different compounds are listed in Supplementary Table S4. All data were collected and acquired by using MassLynx 4.1 (Waters) and processed using TargetLynx 4.1 (Waters).

## 3. Results and Discussion

### 3.1. Food Supplement Labeling

Prior to any analysis, the labels of the dietary food supplements were screened for the presence of a batch number, expiration date, amount of total monacolin or monacolin K present in the sample, and in case of the latter also the mandatory warning [45,46]. As can be seen in Table 3, one sample (sample 13) did not mention any lot number and three other samples (samples 3, 6, and 14) did not display the proper warnings on the label. According to commission regulation 2022/860 of the European Parliament and the Council, the following warning needs to be present on the label of RYR products containing monacolins [21]: “Should not be consumed by pregnant or lactating women, children below 18 years old and adults above 70 years old”; “Seek advice from a doctor on consumption of this product if you experience any health problems”; “Should not be consumed if you are taking cholesterol-lowering medication”; “Should not be consumed if you are already consuming other products containing red yeast rice”. Remarkably, these four samples originated from e-commerce platforms that claimed to be based in Belgium, Germany, Ireland, and the UK. All of the samples that were purchased from online registered pharmacies were compliant with these parameters. 

Next, the mandatory labeling of the amount of monacolins present in the samples was assessed. For the majority of the food supplements obtained through e-commerce platforms, the amount of monacolin present was expressed as the amount of total monacolin K (MK and MKA). For samples 14 and 17, it was expressed in the total amount of monacolins, as it should be according to the commission regulation [21]. Samples 10 and 11, on the other hand, did not mention the amount of monacolins present and were in fact devoid of them. This was also the case for samples 1, 15, and 16, although they clearly stated on the label that they were devoid of any monacolin K. In contrast to the above, the majority of the food supplements originating from online pharmacies mentioned their total amount of monacolins, except for samples 28, 31, 34, and 35, where the amount of monacolin K was mentioned. All samples claimed an amount of either total monacolin K (MK_total_) or total monacolins inferior to 3 mg upon consumption of the maximum serving size.

### 3.2. Screening for Synthetic Statins

Previously, chemical analysis of RYR products demonstrated the occurrence of various monacolins [6,7,8,22,47]. Indeed, in addition to MK and MKA, mevastatin, dehydromonacolin K (DMK), and the minor abundant monacolin J (MJ), monacolin L, monacolin M, and monacolin X, either in acid or lactone form, have also been demonstrated to be present in RYR supplements [6,7,47,48]. However, it was recently reported that small amounts of simvastatin could be detected in RYR supplements [22]. These findings are quite surprising and could be due to the previously unreported synthetic capability of the fungus. A biocatalytic process starting from MJ and a biotransformation of MK into simvastatin has been described for the filamentous fungus *Aspergillus terreus* [22,49]. Alternatively, the presence of simvastatin might be the result of some form of adulteration [22]. The latter would not be so surprising as the European Know-X database on falsified medicinal products, including medicines in disguise, reported the occurrence of synthetic statins in dietary supplements. For these reasons, the dietary supplements were screened for the different monacolins present in RYR (MK, MKA, mevastatin, DMK, MJ) and non-RYR statins, for simplicity termed “synthetic statins” (e.g., simvastatin, pravastatin, and the different synthetic statins, including atorvastatin, cerivastatin, fluvastatin, pitavastatin, and rosuvastatin).

As can be seen in Table 3, no synthetic statins were identified in the samples, and the majority of the samples that contained detectable amounts of monacolins were positive for MK, MKA, and DMK. Occasionally, traces of mevastatin were also seen in some samples. These findings suggest that none of the samples were adulterated with synthetic statins other than lovastatin. In contradiction with the previous finding of Rhigetti et al. [22], we did not detect any simvastatin in the samples. This could be due to the higher limit of detection of our methodology (LOD = 25 ng/g) compared to the study from 2021, where they claimed an LOD of 0.01 ng/g and a limit of quantification (LOQ) of 12.5 ng/g. However, the amount of simvastatin ranged into the microgram range per day for the majority of the samples, which should also be detectable with our methodology. 

### 3.3. Citrinin, Total Monacolin K (MK_total_) Content, and Possible Adulteration

During the fermentation of rice with *Monascus purpureus*, secondary metabolites such as citrinin and monacolin K are produced. The mycotoxin citrinin has been shown to result in detrimental health consequences for humans and animals, including nephrotoxicity and hepatotoxicity. Moreover, it has been shown that citrinin is fetotoxic in mice, while rats exposed to high doses of citrinin develop renal tumors and suffer teratogenic effects [50]. For this reason, the European Commission limited the daily intake of citrinin and, in 2014, specifically limited the amount of citrinin allowed in red yeast rice products to a maximum of 2000 µg/kg food supplement [51]. However, based on new occurrence data and the risk–benefit ratio of red yeast rice supplements, this limit was lowered in 2020 to 100 µg/kg [26]. The history of monacolin is somewhat similar. In 2011, the European Food and Safety Authority (EFSA) approved a health claim for monacolin K from red yeast rice [52]. The panel concluded that a positive health effect for adults could be attributed to RYR, provided that 10 mg of monacolin K was consumed daily, to contribute to the maintenance of normal blood LDL-cholesterol levels. However, in 2018, the EFSA published a report on the safety of monacolins from RYR, and reported that there were safety concerns with dosages of 3 mg/day or higher [7]. That opinion concluded that “due to the composition of RYR and in particular: the presence of monacolin K (also called lovastatin when marketed as a drug) that shares the adverse effects of statins; the presence at varying levels of the other monacolins, compounds whose safety has not been established, consumption of “red yeast rice” exposes some consumers to a health risk” [21,26]. Subsequently, in 2022 the European Commission reduced the maximum allowed daily dosage of total monacolins from RYR supplements to 3 mg/day [21]. As MK and MKA are the major monacolins present in RYR supplements, often representing at least 75% of the total monacolin content [7,48], and labeled reference standards were available, it was decided for this study to focus on their quantification. The samples were analyzed by means of a newly developed methodology to simultaneously quantify citrinin, MK, and MKA. This new methodology employed liquid chromatography coupled to high-resolution accurate mass (LC-HRAM) mass spectrometry (MS) and the usage of stable isotope labeled internal standards (see Materials and Methods and Table 1). The methodology was subsequently validated according to ISO 17025, by employing the total error approach and accuracy profiles (see Supplemental Table S3 and Supplemental Figure S1). The acceptance limits were set at [−20%; 20%] due to the nature of the matrix (herbal extracts and herbal mixtures).

Both the quantification results of citrinin and MK_total_ (MK and MKA) are listed in Table 3. Citrinin was detected in 16 of the 35 samples, indicating that 19 samples contained less than 5 ng of this molecule per gram of material. This value is 20 times lower than the current maximum tolerated amount of citrinin present in RYR supplements in Europe [26]. From these 16 samples, citrinin could be quantified in 9 of the 35 samples (7 samples had a value below the limit of quantification). The found values per maximum serving size varied from 21.4 ± 0.1 µg/kg to 58.7 ± 0.3 µg/kg and were thus all below the tolerated maximum daily intake. MK and MKA were detected in 29 samples. Of the six samples that were devoid of MK and/or MKA, only three mentioned the absence of MK on their label (see Table 3). The amount of MK_total_ was determined for the maximum serving size; it ranged from 0.81 ± 0.01 mg to 6.13 ± 0.12 mg. The latter values exceed the maximum tolerated amount by a factor two. In fact, 3 out of the 35 samples contained a value of MK_total_ that exceeded 3 mg per maximum serving size, taking into account the calculated measurement uncertainty (see Table 3). Sample 14 contained 3.51 ± 0.06 mg, sample 18 contained 4.25 ± 0.21 mg, and sample 31 contained 6.13 ± 0.12 mg of MK_total_. These samples are thus non-compliant with European legislation.

Although the utilized approach is not able to quantify every monacolin, it can already be used during control programs to eliminate those samples/batches that contain more than 3 mg of MK_total_. Ideally, when a labeled standard for both dehydromonacolin (DMK) and Dihydromonacolin K (DiMK) would be available for reasonable financial retribution, it might be worthwhile to include the quantification of these molecules, especially since a recent study has shown that the sum of MK_total_, DMK, and DiMK represents at least 90% of total monacolin content [48]. 

In 2015, Nannoni et al. [30] suggested, based on their analysis of several RYR extracts, that the amount of MK in non-adulterated RYR samples did not exceed 70% of the MK_total_. This can be reformulated as follows: the amount of MKA divided by MK_total_ should result in a number exceeding 0.3, to be certain to exclude possible adulteration. Applying this ratio to the found quantities (see Table 4) resulted in 11 samples with a value below 0.3. This suggests that these samples might be subjected to adulteration by the addition of lovastatin, obtained by the production of lovastatin by *Aspergillus terreus*, which is cheaper [52]. In order to determine if an adulteration with lovastatin from *A. terreus* took place, 10 of the 11 samples were subjected to EA-IRMS to measure their stable carbon isotope ratio (δ13C) values. The δ^13^C was not determined for sample 21 as the sample did not consist of powder but of an oily matrix for which the utilized methodology has not been tested yet. 

Recent studies have shown that the application of this technique is able to distinguish monacolin K from lovastatin in RYR supplements as the respective molds *M. purpureus* and *A. terreus* use different plant sources with different photosynthetic strategies for their fermentation [32,53,54]. Rice, used by *Monascus* species, and sugar cane or corn, used by *A. terreus*, are C3 and C4 plants, respectively, using different photosynthetic strategies that result in unique, non-overlapping δ^13^C distributions [54]. The work performed by Perini et al., enabling the detection of a minimum of 10% MK being produced in *A. terreus*, determined the range of δ^13^C values of monacolin K and lovastatin to be −30.7‰ to −28.2‰ and −21.6‰ to −13.9‰, respectively [32]; thus, values greater than −28‰ are indicative of adulteration. From the results shown in Table 4, it can be seen that 3 samples from the 11 samples exceeded this δ^13^C value, suggesting that these samples contained, at least partly, lovastatin not produced by the fermentation of rice. 

### 3.4. Bioburden, Identification of Micro-Organisms, and Mycotoxin Determination

A recent systematic review, performed for herbal medicinal products, demonstrated that quite often these herbal products suffered from microbial contamination that exceeded the regulatory limits [55]. Although there are no official maximum limits set for micro-organisms that might be present in herbal-derived food supplements, the limits defined in the European Pharmacopoeia (Ph. Eur.) for herbal medicinal products are often used as a quality criterium [33,34]. In the USA, on the other hand, the United States Pharmacopoeia (USP) has a chapter devoted to nutritional and dietary supplements from herbal origin [32]. In the USP, seven different categories are recognized, including dried or powdered botanicals, such as RYR. For this category, the maximum acceptable bacterial load corresponds to 10^5^ colony forming units (CFU) per gram or mL material, and maximum 2000 CFU/g or mL of yeasts and molds can be present. Moreover, the number of micro-organisms that are tolerant to bile acids should be less than 2000 CFU/g or mL and they should be devoid of *Salmonella* spp., *Escherichia coli, Staphylococcus aureus*, and *Clostridium* spp. [32]. The Ph. Eur., on the other hand, describes three different threshold limits in the function of the treatment method of the medicinal herbal product: (i) method A if the medicinal herbal product is to be treated with boiling water; (ii) a default “method B”; and (iii) “method C”, applicable to products where the pre-treatment or processing is not able to reduce the load in micro-organisms and should comply to the limits described in “method B” [32]. For method B, the maximum acceptable bacterial load corresponds to 50,000 CFU/g or mL, while a maximum 500 CFU/g of yeasts and molds can be present. Moreover, the number of micro-organisms that are tolerant of bile acids should also be less than 200 CFU/g and they should be devoid of *Salmonella* spp. or *E*. *coli.* Method C tolerates up to 500,000 CFU/g or mL of bacteria, 50,000 CFU/g or mL of yeasts and molds, and 20,000 CFU/g or mL of bile acid-tolerant bacteria and requires the absence of *Salmonella* spp. or *E*. *coli* [32]. 

From the production process of RYR supplements, it is impossible to declare if these food supplements should either be compliant with method B or method C. Nevertheless, these supplements were analyzed for their total aerobic microbial count, their total yeast and mold count, and the absence of *Salmonella* spp. or *E*. *coli.* The encountered bacterial and fungal species were identified. As can be seen in Table 5, 14 of the 35 samples contained at least 50 CFU/g or mL of bacteria, while only 4 samples contained at least 50 CFU/g or mL of fungi. The bacterial load ranged from 100 to 35,000 CFU/g or mL, while the number of fungi ranged from 50 to 150 CFU/g or mL. Despite the presence of bacteria or fungi, these samples were thus compliant with the requirement of both the USP for dietary supplements and method B as mentioned in the Ph. Eur. for herbal medicinal products. Furthermore, no *Salmonella* spp. or *E*. *coli* was detected. However, in sample 29, approximately 2500 CFU/g or mL of bile acid-tolerant bacteria were found, exceeding the limits of the USP and the Ph. Eur. in case of the application of method B. The application of method B makes sense based on the results obtained for the other samples. Next, identifications were performed of the encountered bacteria and fungi. The majority of the samples with a bacterial load were positive for a species from the *Bacillus* genus (see Table 5). This genus has often been encountered in food supplements and its presence can be associated with the production of endospores resistant to unfavorable physical conditions, such as high temperatures and low humidity. Therefore, they can survive long periods in a hibernating condition on a product. Most species of the genus *Bacillus* are non-pathogenic upon oral uptake; however, the species *Bacillus cereus* has been associated with foodborne illnesses [56,57]. Only sample 20 of the sample set was contaminated with *B. cereus* and this to an extent of 4 × 10^2^ CFU/g. 

In addition to *Bacillus, Niallia taxi*, *Roseomans mucosa*, *Kocuria rhizophila,* and *Acinetobacter johnsonii* were also encountered in the samples. Species belonging to the *Niallia* genus previously belonged to the *Bacillus* genus and are also able to produce resistant endospores [58]. Little to no information is available on the found species, except that it was isolated from the root of *Taxus chinensis*. *R. mucosa* are environmental bacteria and infections in humans are mainly limited to catheter-related bloodstream infections [59]. *Kocuria rhizophila* is present in various environments and is often found in food [60]. These bacteria are able to harbor a multitude of antibiotic-resistance genes. *Acinetobacter johnsonii* has been found in both dry and moist environments and has been reported as an opportunistic pathogen [61]. Genetic analysis of the different *A. johnsonni* isolates found that these bacteria are also able to carry different antibiotic-resistance genes. At least to our knowledge, none of the abovementioned strains are reported to be often associated with foodborne infections, although it is known that *Acinetobacter* sp. are able to colonize the digestive tract and can result in outbreaks amongst vulnerable individuals [61]. Moreover, the occurrence of *K. rhizophilia* and *A. johnsonni* might be considered undesired as these species could act as a potential reservoir of antibiotic-resistance genes which could be transferred to other clinically relevant micro-organisms [60,61]. Nevertheless, the amount of *A. johnsonii* present in sample 29 exceeds the limits set by the Ph. Eur. and by the USP and can thus be considered a potential risk for the safety of the consumer.

In addition to bacteria, the encountered fungi were also identified (see Table 5). The species identified belonged either to the genus *Aspergillus*, *Penicillium*, or *Cladosporium* and did not correspond to any of the species frequently used in the commercial production of lovastatin. Nevertheless, as few foodborne fungi cause gastro-intestinal infections, it is mainly the amount of mycotoxins, especially the carcinogenic ochratoxins and aflatoxins produced by *Aspergillus* sp. and *Penicillium* sp., that are important from a food safety point of view. 

For this reason, the samples were analyzed for the occurrence of the aflatoxins (AFB1, AFB2, AFG1, and AFG2), ochratoxin A (OTA), trichothecene mycotoxins T-2 and HT-2, deoxynivalenol (DON), fumonisins (FB1, FB2, and FB3), and zearalenone (ZEN). As can be seen in Table 6, none of the samples contained quantifiable amounts of aflatoxins. Only one sample, sample 5, contained quantifiable amounts of OTA, although at concentrations that corresponded to one-third of the maximum permitted OTA concentration that is tolerated in dried spices, which are used in mass quantities similar to the mass of the dietary supplements [62]. That specific sample also contained T2, HT2, and DON in quantities below the respective acceptable daily intake values, taking into account a body weight of 60 kg [63,64]. The occurrence of these toxins could not be simply explained by the presence of *A. niger* in the sample and might be the result of contamination with other fungi. The 13 other samples that contained at least one measurable amount of the abovementioned toxins contained toxins in concentrations below 1% of the ADI for a person with a body weight of 60 kg [63,64,65,66]. 

## 4. Conclusions

In this work, different quality parameters which could play an important role in guaranteeing the safety of RYR dietary supplements were assessed. A total of thirty-five RYR supplements, either purchased from an EU-bound e-commerce platform or from registered online pharmacies, were screened for the presence of different monacolins, citrinin, and non-RYR-related statins. The amount of MK_total_ and citrinin was determined. None of the RYR samples contained synthetic statins, and all of the samples were compliant with their citrinin content. However, three supplements contained more than 3 mg of monacolins, and three other samples are suspected of being adulterated with lovastatin. 

Next, the amount of other mycotoxins and the bacterial load were assessed. None of the samples contained quantifiable amounts of one of the four aflatoxins and none of the quantities of mycotoxins that were encountered were reason for concern. The bioburden analysis showed that one sample was severely contaminated with an *Acinetobacter* species that was able to grow on bile acid medium. The presence of more than 2.5 × 10^3^ CFU makes this sample non-compliant with the quality requirements set out by the United States Pharmacopoeia for dietary supplements and those set by the European Pharmacopoeia for herbal medicinal products. Although the latter is specifically dedicated to medicinal products, very often the described limits are used as quality control limits for dietary supplements.

Taken together, our analytical data demonstrate that at least four of the thirty-five RYR supplements (≈11%) might be reason for concern for the safety of the consumer, either due to high monacolin concentrations or bacterial contamination. Moreover, three samples (≈9%) were likely adulterated and the labeling of six of the seventeen samples (≈35%) originating from EU-based e-commerce platforms was not compliant, as either the mandatory warning was missing or incomplete or the amount of monacolins was not mentioned.

## Figures and Tables

**Table 1:** Summary of the LC-MS/MS characteristics and the performance characteristics for quantification of the three different compounds. Abbreviation: D3 = trideuterated compound, LLOQ = lower limit of quantification, ULOQ = upper limit of quantification, SIL = stable isotope labeled (C13).

	Retention Time (min)	Precursor Ion (*m*/*z*)	Fragment Ions (*m*/*z*) and Their Relative Intensities	Limit of Quantification (LLOQ)	Linear Range (LLOQ-ULOQ)
citrinine	3.4	251.091 [M + H]^+^	91.054 (100), 119.085 (50)	5 ng/mL (20 ng/g food supplement)	5–1000 ng/mL
SIL-citrinine	3.4	264.1339 [M + H]^+^	98.0781 (100), 128.1159 (70)		
MKA	4.0	405.262 [M - H_2_O + H]^+^	199.148 (100), 173.132 (80)	100 ng/mL (500 ng/g food supplement)	100–5000 ng/mL
D3-MKA	4.0	408.282 [M - H_2_O + H]^+^	199.148 (100), 173.132 (90)		
MK	4.5	405.262 [M - H_2_O + H]^+^	199.148 (100), 173.132 (75)	50 ng/mL (250 ng/g food supplement)	50–2000 ng/mL
D3-MK	4.5	408.282 [M - H_2_O + H]^+^	199.148 (90), 173.132 (100)		

**Table 2:** Summary of the obtained recoveries for different concentrations in different matrices. Matrix 1: mixture of olive extract, *Allium sativum* extract, and *Allium ursinum* extract; Matrix 2: mixture of Bergamot extract, artichoke extract, and vitamin C; Matrix 3: mixture of coenzyme Q10, olive extract, Pterocarpus extract, vine extract, vitamin B1, vitamin C, vitamin E, and vitamin K. Matrix 4: Red yeast rice sample devoid of detectable amounts of MK and MKA.

	Concentration Level	Recovery (%)		
		Citrinine	MKA	MK
Matrix 1	LLOQ	93.2	104.4	96.5
	ULOQ	102.8	104.7	96.7
Matrix 2	LLOQ	98.3	96.3	100.5
	ULOQ	97.5	97.9	98.4
Matrix 3	LLOQ	92.6	91.5	98.5
	ULOQ	97.2	105.6	94.8
Matrix 4	LLOQ	n.a.	94.5	103.7
	ULOQ	n.a.	98.6	95.6

**Table 3:** Information provided on the label and actual content of citrinin and total monacoline K (MK and MKA) present in the different dietary supplements.

Sample N°	Lot Number and Expiration Date Present	Warning on the Label ^a^	Ingredients or Other API Declared on the Label	Maximum Serving Size According to Labeled Directions	Labeled Amount (mg) per Maximum Serving Size		Synthetic Statins Found in the Sample ^c^	Amount of Citrinin Found (Mean ± MU, ng/g)	Measured Amount (mean ± MU, mg) per Maximum Serving MK_total_
					Total Monacolin	MK_total_ ^b^			
1	yes	n.a.	n.d.	2 capsules	absent	absent	n.d.	46.2 ± 0.3	n.d.
2	yes	yes	Coenzyme Q10 and phytosterols	3 capsules	n.d.	2.8	n.d.	n.d.	2.36 ± 0.1
3	yes	no	Leucine	1 capsule	n.d.	2.95	n.d.	n.d.	1.79 ± 0.02
4	yes	yes	Coenzyme Q10, vitamin E, vitamin C, niacin, plant extracts (sage, garlic, *Astragalus*, sugar cane)	1 capsule	n.d.	2.54	n.d.	n.d.	1.19 ± 0.03
5	yes	yes	Coenzyme Q10, vitamin B6, plant extracts (*Silybum* and *Filipendula*)	1 capsule	n.d.	2.9	n.d.	n.d.	1.54 ± 0.03
6	yes	no	n.d.	1 capsule	n.d.	2.9	n.d.	n.d.	2.78 ± 0.12
7	yes	yes	Leucine	1 capsule	n.d.	2.95	n.d.	n.d.	2.57 ± 0.04
8	yes	yes	n.d.	1 capsule	n.d.	1.0	n.d.	40.5 ± 0.6	1.15 ± 0.04
9	yes	yes	Coenzyme Q10, vitamin B3	1 tablet	n.d.	2.9	n.d.	n.d.	2.15 ± 0.06
10	yes	n.a.	n.d.	2 tablets	n.d.	n.d.	n.d.	58.7 ± 0.3	n.d.
11	yes	n.a.	Coenzyme Q10	2 soft gels	n.d.	n.d.	n.d.	<20	n.d.
12	yes	yes	Vitamin B1	1 capsule	n.d.	2.9	n.d.	n.d.	1.13 ± 0.02
13	no	no	n.d.	2 capsules	n.d.	n.d.	n.d.	n.d.	n.d.
14	yes	no	Coenzyme Q10	2 capsules	2.4	n.d.	n.d.	<20	3.51 ± 0.06
15	yes	no	n.d.	2 capsules	n.d.	absent	n.d.	23.8 ± 0.1	n.d.
16	yes	no	n.d.	2 capsules	n.d.	absent	n.d.	24.0 ± 0.04	n.d.
17	yes	yes	n.d.	1 capsule	2.9	n.d.	n.d.	<20	2.66 ± 0.02
18	yes	yes	n.d.	1 tablet	2.99	n.d.	n.d.	<20	4.25 ± 0.24
19	yes	yes	Folic acid, coenzyme Q10, berberine	1 tablet	2.8	n.d.	n.d.	n.d.	1.84 ± 0.05
20	yes	yes	Coenzyme Q10, folic acid, vitamin B12, plant extract (*Coriandrum sativum*)	1 tablet	2.9	n.d.	n.d.	41.9 ± 0.2	1.97 ± 0.05
21	yes	yes	Coenzyme Q10, different plant extracts (*Allium sativum* and *Olea europeae*)	2 liquid capsules	2.99	n.d.	n.d.	21.4 ± 0.1	1.52 ± 0.02
22	yes	yes	Coenzyme Q10, different plant extracts (*Allium sativum* and *Olea europeae*)	2 capsules	2.99	n.d.	n.d.	n.d.	1.31 ± 0.05
23	yes	yes	n.d.	1 capsule	2.9	n.d.	n.d.	n.d.	1.41 ± 0.04
24	yes	yes	Coenzyme Q10 and berberine	2 capsules	2.99	n.d.	n.d.	n.d.	3.19 ± 0.2
25	yes	yes	Vitamin B1, plant extracts (*Cynara scolymus* and *Olea europeae*)	1 tablet	2.9	n.d.	n.d.	n.d.	1.30 ± 0.15
26	yes	yes	Plant extracts (*Cynara scolymus* and *Olea europeae*)	1 tablet	2.95	n.d.	n.d.	n.d.	1.25 ± 0.03
27	yes	yes	Coenzyme Q10, cinnamon and green tea extract	½ tablet	2.885	1.9	n.d.	33.6 ± 0.2	0.81 ± 0.01
28	yes	yes	Vitamin B3, vitamin E, and coenzyme Q10	1 tablet	n.d.	2.99	n.d.	n.d.	1.58 ± 0.03
29	yes	yes	n.d.	1 tablet	2.5	n.d.	n.d.	38.7 ± 0.1	2.31 ± 0.07
30	yes	yes	Vitamin B1	1 tablet	2.9	n.d.	n.d.	<20	1.83 ± 0.07
31	yes	yes	Ascorbic acid, vitamin B1, different plant extracts (sage, *Coriandrum sativum*, and *Tinospora cordifolia*)	2 capsules	n.d.	1.48	n.d.	n.d	6.13 ± 0.12
32	yes	yes	n.d.	1 capsule	2.95	n.d.	n.d.	n.d	2.71 ± 0.04
33	yes	yes	Plant extract (*Allium sativum* and *Cynara scolymus*)	1 capsule	2.99	n.d.	n.d.	n.d.	0.99 ± 0.03
34	yes	yes	n.d.	1 capsule	n.d.	2.95	n.d.	<20	2.53 ± 0.09
35	yes	yes	n.d.	1 capsule	n.d.	2.9	n.d.	<20	2.34 ± 0.04

^a^ According to commission regulation 2022/860 of the European parliament and the Council as regards monacolins from red yeast rice, the following warning needs to present on the label of RYR products containing monacolins: “Should not be consumed by pregnant or lactating women, children below 18 years old and adults above 70 years old”; “Seek advice from a doctor on consumption of this product if you experience any health problems”; “Should not be consumed if you are taking cholesterol-lowering medication”; “Should not be consumed if you are already consuming other products containing red yeast rice”. ^b^ MK_total_ = the sum of the amount of MKA and MK. ^c^ These statins are either produced naturally or synthetically, but are not reported to be synthesized by *M. purpureus*. Pravastatin is the result of a biotransformation process by micro-organisms such as *Streptomyces* spp. or *Actinomadura* spp., while simvastatin is synthesized by *Aspergillus terreus* and *Penicillium citrinum*. The screening detection limit of the different statins can be found in Supplemental Table S2. Abbreviations: MK = monacolin K; MKA, monacolin K hydroxyacid; n.a. = not applicable; n.d. = not detected; MU = uncertainty of measurement. Citrinin was also included in the screening methodology. The newly developed LC-HRAM MS screening methodology was able to distinguish citrinin, the different monacolins, and statins from each other and from the different matrix ingredients. The specificity and the selectivity of the method were demonstrated at the screening detection limit (SDL) for the different compounds (see Supplemental Table S2). The SDL corresponds to the lowest concentration where the signal-to-noise (S/N) ratio (peak to peak) reached a value equal to or exceeding 3.3 and where the fragment ions were still present in the MS^n^ spectra.

**Table 4:** The amount of MK, MKA, and MK_total_; the ratio MKA to MK_total_; and the δ^13^C found in the different monacolin K-containing samples. The MU is expressed as a confidence interval using the standard deviation of the generated quantification results.

Sample N°	Amount Found per Maximum Serving Size (mean ± MU, mg)			Ratio MKA/MK_total_	δ^13^ C Values of MK (‰)
	MKA	MK	MK_total_		
2	0.70 (±0.03)	1.66 (±0.02)	2.36 (±0.09)	0.30	n.a.
3	1.35 (±0.03)	0.44 (±0.02)	1.79 (±0.03)	0.75	n.a.
4	0.15 (±0.01)	1.04 (±0.03)	1.19 (±0.03)	0.13	−17.6
5	0.70 (±0.03)	0.84 (±0.01)	1.54 (±0.03)	0.45	n.a.
6	2.17 (±0.12)	0.61 (±0.003)	2.78 (±0.12)	0.78	n.a.
7	1.95 (±0.03)	0.61 (±0.03)	2.56 (±0.04)	0.76	n.a.
8	0.18 (±0.01)	0.96 (±0.03)	1.15 (±0.04)	0.16	−30.1
9	0.88 (±0.02)	1.26 (±0.03)	2.15 (±0.06)	0.41	n.a.
12	0.56 (±0.03)	0.58 (±0.01)	1.13 (±0.02)	0.49	n.a.
14	0.99 (±0.02)	2.52 (±0.05)	3.51 (±0.06)	0.28	−28.5
17	0.63 (±0.01)	2.02 (±0.02)	2.66 (±0.02)	0.24	−28.1
18	3.67 (±0.25)	0.58 (±0.01)	4.25 (±0.24)	0.86	n.a.
19	1.23 (±0.03)	0.61 (±0.02)	1.84 (±0.05)	0.67	n.a.
20	0.23 (±0.01)	1.73 (±0.05)	1.97 (±0.05)	0.12	−29.6
21	0.41 (±0.02)	1.11 (±0.03)	1.52 (±0.02)	0.27	n.d.
22	0.43 (±0.03)	0.88 (±0.01)	1.31 (±0.05)	0.33	n.a.
23	0.42 (±0.04)	0.99 (±0.02)	1.41 (±0.04)	0.30	n.a.
24	1.15 (±0.05)	2.03 (±0.03)	3.19 (±0.20)	0.36	n.a.
25	0.51 (±0.01)	0.79 (±0.02)	1.3 (±0.02)	0.39	n.a.
26	0.35 (±0.03)	0.91 (±0.04)	1.25 (±0.03)	0.28	−22.6
27	0.44 (±0.02)	0.37 (±0.01)	0.81 (±0.01)	0.54	n.a.
28	1.04 (±0.05)	0.54 (±0.02)	1.58 (±0.03)	0.66	n.a.
29	0.72 (±0.03)	1.58 (±0.06)	2.3 (±0.07)	0.31	n.a.
30	0.93 (±0.01)	0.90 (±0.02)	1.83 (±0.07)	0.51	n.a.
31	5.97 (±0.06)	0.16 (±0.004)	6.13 (±0.12)	0.97	n.a.
32	0.39 (±0.01)	2.31 (±0.05)	2.7 (±0.04)	0.14	−30.0
33	0.22 (±0.02)	0.77 (±0.04)	0.99 (±0.03)	0.22	−25.0
34	0.56 (±0.03)	1.96 (±0.12)	2.52 (±0.09)	0.22	−30.4
35	0.56 (±0.03)	1.78 (±0.06)	2.34 (±0.04)	0.24	−30.2

Abbreviations: n.a. = not applicable; n.d. = not detected.

**Table 5:** Microbial load and the micro-organisms identified by MALDI-TOF.

Sample N°	TAMC		Growth on Bile Acid Medium (CFU/g or mL)	TYMC	
	CFU/g or mL	Identified Organism		CFU/g or mL	Identified Organism
1	n.d.	n.d.	n.d.	n.d.	n.d.
2	2.5 × 10^3^	*Bacillus velenzis*	n.d.	n.d.	n.d.
3	n.d.	n.d.	n.d.	50	*Penicillium* *olsonii*
4	2 × 10^3^	*Bacillus velenzis* and *Bacillus* spp.	n.d.	n.d.	n.d.
5	2.5 × 10^2^	*Bacillus velenzis*	n.d.	50	*Aspergillus niger*
6	1 × 10^3^	*Bacillus* spp.	n.d.	n.d.	n.d.
7	n.d.	n.d.	n.d.	n.d.	n.d.
8	n.d.	n.d.	n.d.	n.d.	n.d.
9	n.d.	n.d.	n.d.	n.d.	n.d.
10	2 × 10^2^	*Bacillus subtilis*	n.d.	n.d.	n.d.
11	n.d.	n.d.	n.d.	n.d.	n.d.
12	n.d.	n.d.	n.d.	n.d.	n.d.
13	1.5 × 10^2^	*Roseomonas mucosa*	n.d.	n.d.	n.d.
14	3.5 × 10^4^	*Bacillus subtilis*	n.d.	n.d.	n.d.
15	n.d.	n.d.	n.d.	n.d.	n.d.
16	3 × 10^2^	*Kocuria rhizophila*	n.d.	n.d.	n.d.
17	1.5 × 10^3^	*Bacillus* spp. and *Staphylococcus capitis*	n.d.	n.d.	n.d.
18	n.d.	n.d.	n.d.	n.d.	n.d.
19	n.d.	n.d.	n.d.	n.d.	n.d.
20	4 × 10^2^	*Bacillus cereus*	n.d.	n.d.	n.d.
21	n.d.	n.d.	n.d.	n.d.	n.d.
22	2 × 10^2^	*Bacillus amyloliquifaciens*	n.d.	n.d.	n.d.
23	n.d.	n.d.	n.d.	n.d.	n.d.
24	n.d.	n.d.	n.d.	n.d.	n.d.
25	n.d.	n.d.	n.d.	n.d.	n.d.
26	n.d.	n.d.	n.d.	n.d.	n.d.
27	50	*Niallia taxi*	n.d.	n.d.	n.d.
28	n.d.	n.d.	n.d.	n.d.	n.d.
29	2.5 × 10^3^	*Acinetobacter johnsonii*	2.5 × 10^3^	150	*Cladosporium uwebraunianum*
30	n.d.	n.d.	n.d.	n.d.	n.d.
31	n.d.	n.d.	n.d.	n.d.	n.d.
32	n.d.	n.d.	n.d.	n.d.	n.d.
33	n.d.	n.d.	n.d.	75	*Penicillium rubens*
34	1 × 10^2^	*Bacillus* spp.	n.d.	n.d.	n.d.
35	n.d.	n.d.	n.d.	n.d.	n.d.

Abbreviations: TAMC = total aerobic microbial growth; TYMC = total yeast and mold count; CFU = colony forming units; n.d. = not detected.

**Table 6:** Amount of mycotoxin found in the different samples. The amount of aflatoxins (AF, sum of the amount of AFB1, AFB2, AFG1, and AFG2), ochratoxin A (OTA), trichothecene mycotoxins T-2 and HT-2, deoxynivalenol (DON), fumonisins (FB1, FB2, and FB3), and zearalenone (ZEN) was determined.

Sample N°	Mycotoxin Concentration (ng/g)					
	AF	OTA	T2 + HT2	DON	FB_1–3_	ZEN
1	n.d.	n.d.	n.d.	n.d.	n.d.	96.70
5	n.d.	5.82	724.09	288.25	<10	n.d.
6	n.d.	n.d.	n.d.	412.35	n.d.	n.d.
7	n.d.	n.d.	n.d.	28.05	<10	n.d.
10	n.d.	n.d.	n.d.	n.d.	n.d.	29.06
11	n.d.	n.d.	n.d.	n.d.	n.d.	25.90
13	n.d.	n.d.	n.d.	n.d.	n.d.	16.95
14	<1	n.d.	n.d.	n.d.	17.72	n.d.
16	n.d.	n.d.	n.d.	n.d.	n.d.	65.19
21	n.d.	n.d.	n.d.	n.d.	32.23	n.d.
33	n.d.	n.d.	n.d.	51.57	17.75	n.d.
35	<1	n.d.	n.d.	n.d.	17.41	n.d.
Max. limit (ng/g)	4 ^a^	15 ^b^	-	-	-	-
TDI (ng/kg bw)	-	-	20	1000	2000	250
ADI (ng)	-	-	1200	60,000	120,000	15,000

^a^ The maximum limit mentioned corresponds to the maximum limit set by the European Pharmacopoeia for herbal medicines and corresponds to a total of 4 ng of aflatoxins per gram of herbal medicine. ^b^ The maximum limit mentioned corresponds to the maximum limit set by the European Food Safety Authority for spice mixes as the mass quantities used per day for these food commodities resemble the daily mass quantity of these dietary supplements. Abbreviations: ADI = acceptable daily intake corresponding to the TDI for a person with a mean body weight of 60 kg; TDI = tolerable daily intake; n.d. = not detected.

## Data Availability

The original contributions presented in this study are included in the article/Supplementary Materials, further inquiries can be directed to the corresponding author.

## Supplementary Materials

The following supporting information can be downloaded at https://www.mdpi.com/article/10.3390/foods13121919/s1: Figure S1: Accuracy profiles for MK, MKA, and MK_total_; Table S1: API listed on the label and country of origin of the RYR supplements; Table S2: Overview of the target analytes, their retention time, exact masses, their fragment ion and relative intensities, and the obtained screening detection limit; Table S3: Summary of the validation data of the quantification methodology, including trueness, precision, accuracy, and relative expanded uncertainty; Table S4: MS settings and transitions followed for the different mycotoxins.
