# Supplementary material for: Quality Control and Safety Assessment of Online-Purchased Food Supplements Containing Red Yeast Rice (RYR)
Source: Foods. 2024 Jun 18;13(12):1919. doi: 10.3390/foods13121919 (PMC11202976; doi:10.3390/foods13121919)
Supplement: Supplementary file 1 [file foods-13-01919-s001.zip › foods-3062094-supplementary.pdf]

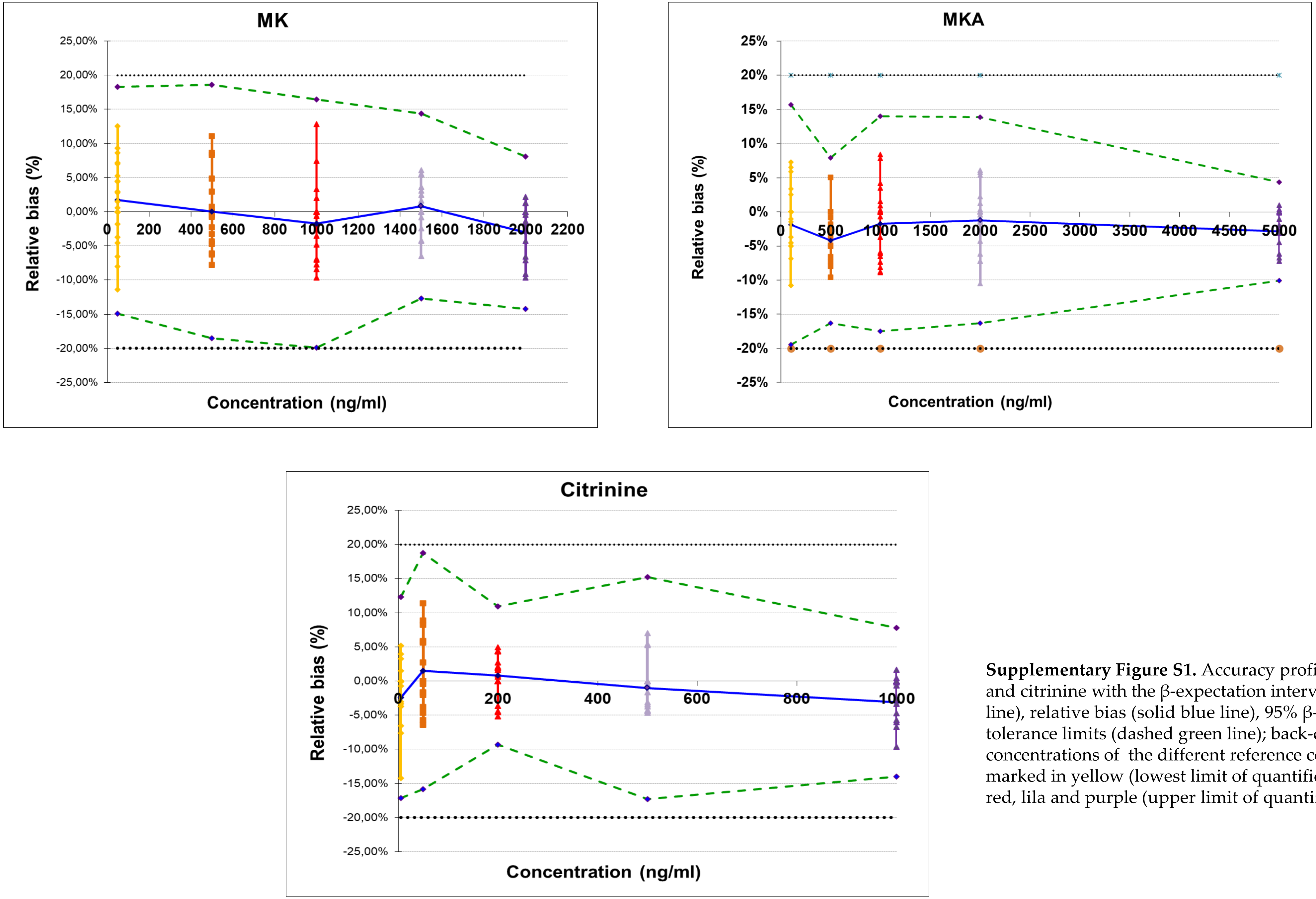

Supplementary Figure S1. Accuracy profiles of MK, MKA and citrinine with the β-expectation interval (black dotted line), relative bias (solid blue line), 95% β-expectation tolerance limits (dashed green line); back-calculated concentrations of the different reference concentrations are marked in yellow (lowest limit of quantification), orange, red, lila and purple (upper limit of quantification).

n°	Other active substances declared on the label	Country of origin
EU-based e-commerce		
**1**	n.a.	Germany
**2**	Coenzyme Q10 and phytosterols	France
**3**	Leucine	Germany
**4**	Coenzyme Q10, vitamin E, vitamin C, niacin, plant extracts (sage, garlic, Astragalus, sugar cane)	Spain
**5**	Coenzyme Q10, vitamin B6, plant extracts (*Silybum* and *Filipendula*)	Spain
**6**	n.a.	Ireland
**7**	Leucine	Germany
**8**	n.a.	France
**9**	Coenzyme Q10, vitamine B3	Spain
**10**	n.a.	The Netherlands
**11**	Coenzyme Q10	The Netherlands
**12**	Vitamin B1	France
**13**	n.a.	UK
**14**	Coenzyme Q10	The Netherlands
**15**	n.a.	The Netherlands
**16**	n.a.	The Netherlands
**17**	n.a.	Belgium
Online registered pharmacies (licensed by Belgian health authorities)		
**18**	n.a.	
**19**	Folic acid, coenzyme Q10, berberine	
**20**	Coenzyme Q10, folic acid, vitamin B12, plant extract (*Coriandrum sativum*)	
**21**	Coenzyme Q10, different plant extracts (*Allium sativum* and *Olea europeae*)	
**22**	Coenzyme Q10, different plant extracts (*Allium sativum* and *Olea europeae*)	
**23**	n.a.	
**24**	Coenzyme Q10 and berberine	
**25**	Vitamin B1, plant extracts (*Cynara scolymus* and *Olea europeae*)	

**26**	plant extracts (*Cynara scolymus* and *Olea europeae*)	
**27**	Coenzyme Q10, cinnamon and green tea extract	
**28**	Vitamin B3, vitamin E and coenzyme Q10	
**29**	n.a.	
**30**	Vitamin B1	
**31**	Ascorbic acid, vitamin B1, different plant extracts (Sage, *Coriandrum sativum* and *Tinospora cordifolia*)	
**32**	n.a.	
**33**	Plant extracts (*Allium sativum* and *Cynara scolymus*)	
**34**	n.a.	
**35**	n.a.	

Supplementary Table S1. Information on the other active substances mentioned on the label of the different dietary supplements coming either from EU-based e-commerce or from online registered pharmacies as required by Belgian law. The country of origin of the products from the e-commerce are also mentioned. N.a. : not applicable (no information found on the label).

Name	CAS n°	Chemical formula	Mono-isotopic mass (Da)	RT (min)	Dominant precursor ion	Two most intense fragment ions (relative intensities, %)	Screening detection limit (ng/g)
**Toxin[a]**							
Citrinin	518-75-2	$C_{13}H_{14}O_5$	250.0841	3.4	251.091 $[M+H]^+$	91.054 (100), 119.085 (50)	5
**Statins naturally present in RYR[b]**							
Monacolin J (MJ)	79952-42-4	$C_{19}H_{28}O_4$	320.1987	3.3	303.195 $[M-H2O+H]^+$	105.069 (100) 143.085 (80)	25
Hydroxyacid monacolin K (MKA)	75225-51-3	$C_{24}H_{38}O_6$	422.2668	4.0	405.262 $[M-H_2O+H]^+$	199.148 (100), 173.132 (75)	125
Mevastatin	73573-88-3	$C_{23}H_{34}O_5$	390.2406	4.2	391.248 $[M+H]^+$	159.116 (100), 185.132 (90)	25
Monacolin K (MK)	75330-75-5	$C_{24}H_{36}O_5$	404.2563	4.5	405.263 $[M+H]^+$	199.148 (100), 173.132 (85)	25
Dehydrolovastatin (DMK)	109273-98-5	$C_{24}H_{34}O_4$	386.2457	5.4	387. 252 $[M+H]^+$	143.085 (100), 199.148 (100)	25
**Statins not naturally present in RYR[c]**							
Pravastatin	81093-37-0	$C_{23}H_{36}O_7$	424.2460	2.9	269.151 ISF^+	167.085 (100), 181.100 (60)	125
Pitavastatin	147511-69-1	$C_{25}H_{24}FNO_4$	421.1689	3.0	422.176 $[M+H]^+$	290.133 (100), 318.128 (60)	25
Rosuvastatin	287714-41-4	$C_{22}H_{28}FN_3O_6S$	481.1683	3.2	482.176 $[M+H]^+$	258.138 (100), 300.150 (45)	125
Atorvastatin	134523-00-5	$C_{33}H_{35}FN_2O_5$	558.2530	3.6	559.260 $[M+H]^+$	250.101 (100), 292.101 (40)	25

Fluvastatin	93957-54-1	$C_{24}H_{26}FNO_4$	411.1846	3.6	394.180 $[M-H_2O+H]^+$	264.117 (90), 274.100 (100)	25
Cerivastatin	145599-86-6	$C_{26}H_{34}FNO_5$	459.2421	3.7	460.249 $[M+H]^+$	356.201 (100), 400.227 (40)	5
Simvastatin	79902-63-9	$C_{25}H_{38}O_5$	418.2719	4.8	419.279 $[M+H]^+$	199.148 (100), 173.132 (80)	25

Supplementalry Table S2. Overview of the target analytes, their name, CAS number, chemical formula, monoisotopic mass, retention time, their observed dominant precursor ion, dominant fragment ions and the established screening detection limit obtained for the different matrices.

a *Monoascus* sp, the fungus used to generate red yeast rice, is able to produce the mycotoxin citrinin during fermentation. The amount of citrinin being produced depends on the fungal species utilised and the growth conditions.

b Although the literature indicates that the highest concentrations of different statins or monacolins encountered in RYR are hydroxyacid lovastatin, lovastatin and dehydrolovastatin, also minor abundant statins such as lovastatin diol lactone and mevastatin are included in the list as they have been shown to be present in small quantities in RYR products.

[C] These statins are either produced naturally or synthetically, but are not reported to be synthesised by *M. purpureus*. Pravastatin is the result of a biotransformation process by microorganisms such as *Streptomyces* sp. or *Actinomadura* sp while simvastatin is synthesised by *Aspergillus terreus* and *Penicillium citrinum*.

		**Citrinine**		**MKA**		**MK**	
		Concentration (ng/mL)	calculated	concentration (ng/mL)	calculated	concentration (ng/mL)	calculated
**Linearity[a] expressed as R^2**		/	0.99986	/	0.99994	/	0.99909
**Trueness**	relative bias (%)	5	2.41	100	1.88	50	1.71
		50	1.47	250	4.19	250	0.05
		200	0.82	500	1.77	500	1.73
		500	1.03	2000	1.23	1000	0.83
		1000	3.12	5000	2.87	2000	3.06
**Precision**	Intermediate precision (%)	5	4.10	100	2.26	50	3.69
		50	2.26	250	2.09	250	1.69
		200	1.71	500	1.48	500	3.36
		500	1.21	2000	1.97	1000	2.39
		1000	1.93	5000	2.13	2000	3.05
	Repeatability (%)	5	5.80	100	6.38	50	6.85
		50	6.20	250	4.13	250	6.37
		200	3.76	500	5.85	500	6.90
		500	4.78	2000	5.4	1000	4.66
		1000	3.74	5000	2.90	2000	4.37
**Accuracy**	ß-expectation tolerance limits (%)	5	**[-17.1**; 12.3]	100	**[-19.4**; **15.7**]	50	[-14.9; 18.3]
		50	[-15.8; **18.8**]	250	[-16.2; 7.9]	250	[-18.5; **18.6]**
		200	[-9.3; 10.9]	500	[-17.5; 14.0]	500	**[-19.9**; 16.5]
		500	[-17.3; 15.2]	2000	[-16.3; 13.9]	1000	[-12.7; 14.4]
		1000	[-14.0; 7.8]	5000	[-10.1; 4.4]	2000	[-14.2; 8.1]
**Uncertainty**	Relative expanded uncertainty (%)	5	12.5	100	13.6	50	15,6
		50	13.5	250	9.1	250	13,9
		200	8.1	500	12.6	500	14.9
		500	10.6	2000	11.7	1000	10.2
		1000	8.2	5000	6.2	2000	9.4

Supplementalry Table S3. Summary of the validation data of the quantification methodology, including Trueness, precision, accuracy and relative expanded uncertainty. [a] R^2 of the linear relationship between the theoretical and measured concentration. Values in bold indicate the lowest and highest values obtained for the ß-expectation tolerance limits.

Mycotoxin	Precursor ion (m/z)	Cone Voltage (V)	Quantifier ion (m/z)	Collision energy (eV)	Qualifier ion (m/z)	Collision energy (eV)
**Aflatoxins**						
AFG2	331,1	45	285,0	30	217,0	40
AFG2 ^{13}C	348,1	32	258,9	30	n.a.	n.a.
AFG1	329,1	34	242,9	27	199,7	41
AFG1 ^{13}C	346,1	40	257,0	29	n.a.	n.a.
AFB2	315,1	48	258,9	27	243,1	37
AFB2 ^{13}C	332,2	44	303,1	24	n.a.	n.a.
AFB1	313,1	44	241,0	37	213,0	44
AFB1 ^{13}C	330,1	36	300,9	26	n.a.	n.a.
**Ochratoxin A**						
OTA	404,2	30	358,0	16	238,9	25
OTA ^{13}C	424,0	20	249,8	28	n.a.	n.a.
**Trichothecene mycotoxins T2 and HT2**						
HT-2	442,0	12	262,9	12	214,9	12
HT-2 ^{13}C	464,4	17	228,9	13	n.a.	n.a.
T-2	484,1	18	304,9	14	214,9	20
T-2 ^{13}C	508,4	19	322,1	13	n.a.	n.a.
**Fumonisins**						
FB1	722,4	50	334,4	39	352,4	35
FB1 ^{13}C	756,7	51	374,2	34	n.a.	n.a.
FB2	706,4	44	336,4	37	318,4	39
FB2 ^{13}C	740,4	63	358,1	36	n.a.	n.a.
FB3	706,4	44	336,4	37	318,4	39
FB3 ^{13}C	739,9	49	358,2	35	n.a.	n.a.
**Deoxynivalenol**						
DON	297,1	20	249,1	10	203,0	14
DON ^{13}C	312,1	18	262,9	10	n.a.	n.a.
**Zearalenone**						
ZEN	319,1	14	283,1	12	187,0	22
ZEN ^{13}C	337,2	16	199,0	22	n.a.	n.a.

Supplementary Table S4. MS-settings and transitions followed for the different mycotoxins.
Abbreviations: AFB1: aflatoxin B1, AFB2: aflatoxin B2, AFG1: aflatoxin G1, AFG2: aflatoxin G2, OTA: ochratoxin A, DON: deoxynivalenol, FB1: fumonisin B1, FB2: fumonisin B2, FB3: fumonisin B3, ZEN: zearalenone, n.a.: not applicable.
